# SuperCam Calibration Targets: Design and Development

**DOI:** 10.1007/s11214-020-00764-w

**Published:** 2020-11-26

**Authors:** J. A. Manrique, G. Lopez-Reyes, A. Cousin, F. Rull, S. Maurice, R. C. Wiens, M. B. Madsen, J. M. Madariaga, O. Gasnault, J. Aramendia, G. Arana, P. Beck, S. Bernard, P. Bernardi, M. H. Bernt, A. Berrocal, O. Beyssac, P. Caïs, C. Castro, K. Castro, S. M. Clegg, E. Cloutis, G. Dromart, C. Drouet, B. Dubois, D. Escribano, C. Fabre, A. Fernandez, O. Forni, V. Garcia-Baonza, I. Gontijo, J. Johnson, J. Laserna, J. Lasue, S. Madsen, E. Mateo-Marti, J. Medina, P.-Y. Meslin, G. Montagnac, A. Moral, J. Moros, A. M. Ollila, C. Ortega, O. Prieto-Ballesteros, J. M. Reess, S. Robinson, J. Rodriguez, J. Saiz, J. A. Sanz-Arranz, I. Sard, V. Sautter, P. Sobron, M. Toplis, M. Veneranda

**Affiliations:** 1 Unidad Asocida UVA-CSIC-CAB, University of Valladolid (UVA), Valladolid, Spain; 2 Institut de Recherche en Astrophysique et Planétologie (IRAP), CNRS, CNES, Université de Toulouse, Toulouse, France; 3 Los Alamos National Laboratory, Los Alamos, NM USA; 4 Niels Bohr Institute (NBI), University of Copenhagen, Copenhagen, Denmark; 5 University of the Basque Country (UPV/EHU), Leioa, Spain; 6 CNRS, Institut de Planetologie et d’Astrophysique de Grenoble (IPAG), Universite Grenoble Alpes, Saint-Martin d’Heres, France; 7 Institut de Minéralogie, de Physique des Matériaux et de Cosmochimie (IMPMC), CNRS, MNHN, Sorbonne Université, Paris, France; 8 Laboratoire d’Etudes Spatiales et d’Instrumentation en Astrophysique, Observatoire de Paris-PSL, CNRS, Sorbonne Université, Université de Paris, Meudon, France; 9 Ingeniería de Sistemas para la Defensa de España S.A. (ISDEFE), Madrid, Spain; 10 Laboratoire d’astrophysique de Bordeaux, CNRS, Univ. Bordeaux, Bordeaux, France; 11 Added Value Solutions (AVS), Elgóibar, Spain; 12 U. Winnipeg, Winnipeg, Canada; 13 Univ Lyon, ENSL, CNRS, LGL-TPE, Univ Lyon 1, 69007 Lyon, France; 14 CIRIMAT, Université de Toulouse, CNRS/UT3/INP, Ensiacet, Toulouse, France; 15 Observatoire Midi-Pyrénées, Toulouse, France; 16 Instituto Nacional de Técnica Aeroespacial, Torrejón de Ardoz, Spain; 17 GeoRessources, Vandoeuvre les Nancy, France; 18 Instituto de Geociencias CSIC, Universidad Complutense de Madrid, Madrid, Spain; 19 Jet Propulsion Laboratory, Pasadena, CA USA; 20 Applied Physics Laboratory, Johns Hopkins University, Laurel, MD USA; 21 University of Malaga (UMA), Málaga, Spain; 22 Centro de Astrobiología-CSIC-INTA, Torrejón de Ardoz, Spain; 23 SETI Institute, Mountain View, CA USA

**Keywords:** Perseverance rover, Jezero crater, LIBS, Raman spectroscopy, Infrared spectroscopy, SuperCam, Calibration

## Abstract

SuperCam is a highly integrated remote-sensing instrumental suite for NASA’s Mars 2020 mission. It consists of a co-aligned combination of Laser-Induced Breakdown Spectroscopy (LIBS), Time-Resolved Raman and Luminescence (TRR/L), Visible and Infrared Spectroscopy (VISIR), together with sound recording (MIC) and high-magnification imaging techniques (RMI). They provide information on the mineralogy, geochemistry and mineral context around the Perseverance Rover.

The calibration of this complex suite is a major challenge. Not only does each technique require its own standards or references, their combination also introduces new requirements to obtain optimal scientific output. Elemental composition, molecular vibrational features, fluorescence, morphology and texture provide a full picture of the sample with spectral information that needs to be co-aligned, correlated, and individually calibrated.

The resulting hardware includes different kinds of targets, each one covering different needs of the instrument. Standards for imaging calibration, geological samples for mineral identification and chemometric calculations or spectral references to calibrate and evaluate the health of the instrument, are all included in the SuperCam Calibration Target (SCCT). The system also includes a specifically designed assembly in which the samples are mounted. This hardware allows the targets to survive the harsh environmental conditions of the launch, cruise, landing and operation on Mars during the whole mission. Here we summarize the design, development, integration, verification and functional testing of the SCCT. This work includes some key results obtained to verify the scientific outcome of the SuperCam system.

## Introduction

The Mars 2020 mission and its Rover, Perseverance, will continue NASA’s efforts in the exploration of Mars and the search for life on that planet (Mustard et al. 2013). Three of the four main objectives of the mission are related to the search of biosignatures on Mars: to study the geology of the planet and its habitability, to look for potential biomarkers or reservoirs where they could be preserved, and to collect documented samples for a future Mars Sample Return Mission (Farley et al. 2020, this issue). Perseverance leverages the architecture of the Curiosity rover (Mustard et al. 2013), and its heritage is extended to part of the payload, with SuperCam being an example (Wiens et al. 2020; Maurice et al. 2020, this issue).

SuperCam is a standoff instrument, designed to be a multianalytical suite of five co-aligned techniques: Laser-Induced Breakdown Spectroscopy (LIBS), Time-Resolved Raman and Luminescence (TRR/L), Visible (VIS) and Infrared (IR) Spectroscopy (VISIR), Remote Micro-Imaging (RMI) and sound recording (MIC). For detailed descriptions of these techniques, see Maurice et al. (2020, this issue) and Wiens et al. (2020, this issue). This suite, with the synergistic use of all of its techniques, makes SuperCam a cornerstone tool for operational decisions regarding the rover’s actions and traverse, as it extends scientific exploration far beyond the range of action of the robotic arm.

**Fig. 1:**
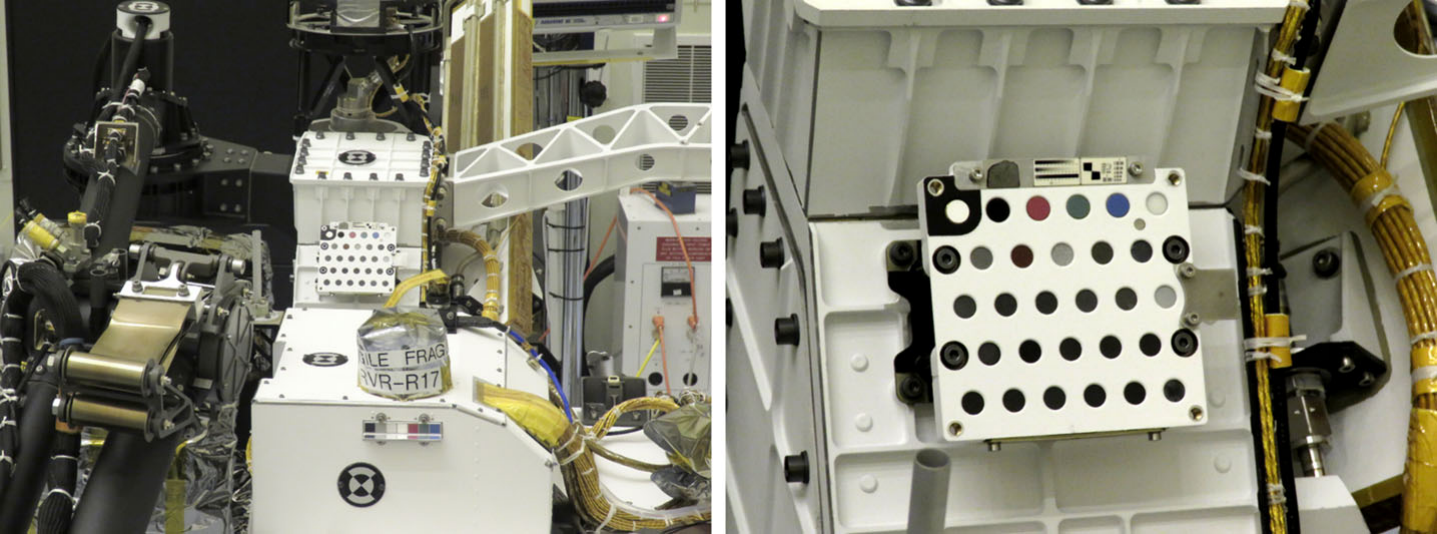
The SuperCam Calibration Targets are at the rear of Perseverance’s deck, on the right side in the forward direction. Photo at Kennedy Space Center. Credits NASA/JPL-Caltech. (Left) An ESD bag is covering the Mastcam-Z primary calibration target; below Mastcam-Z secondary calibration targets

The instrument is composed of three main subsystems: the Body Unit is located inside the body of the rover and contains the three spectrometers used for LIBS, Raman and luminescence, and the VIS range of passive spectroscopy (Wiens et al. 2020, this issue). The Mast Unit is located at the top of the mast, above Mastcam-Z and Navcams, and includes the laser, focusing and collection optics, the microphone, the imager, and the IR spectrometer (Maurice et al. 2020, this issue). The SuperCam Calibration Target (SCCT) assembly is located on the rover deck (Fig. 1), in the same position as the ChemCam calibration target on MSL (Wiens et al. 2012). SuperCam is the result of an international collaboration between Los Alamos National Laboratory (USA) as the leading institution (Roger C. Wiens, instrument PI), IRAP (France), responsible for the Mast Unit (Sylvestre Maurice, Deputy PI), and the Calibration Target, under the coordination of the University of Valladolid (Spain), with Fernando Rull as Co-investigator and science lead for this element.

Besides its standoff capabilities, SuperCam can investigate different features of the target at the same location, including texture, chemistry and structure (mineralogy and organic) composition. In detail, LIBS reveals the elemental composition of the investigated spots, while Raman and passive spectroscopies identify their molecular composition and crystalline structure. Time-resolved luminescence provides information about subtle components in the crystal lattice such as trace elements or the presence of organics. The shock wave generated by the plasma expansion of the laser sparks during LIBS acquisition provides insights about the hardness of the target (Chide et al. 2019), while the high-magnification images put all the previous measurements in a visual and morphological context. The different information coming from each technique allows better characterization of the targets and facilitates the application of multi-technique data fusion (Manrique-Martinez et al. 2020; Moros and Laserna 2015).

**Fig. 2:**
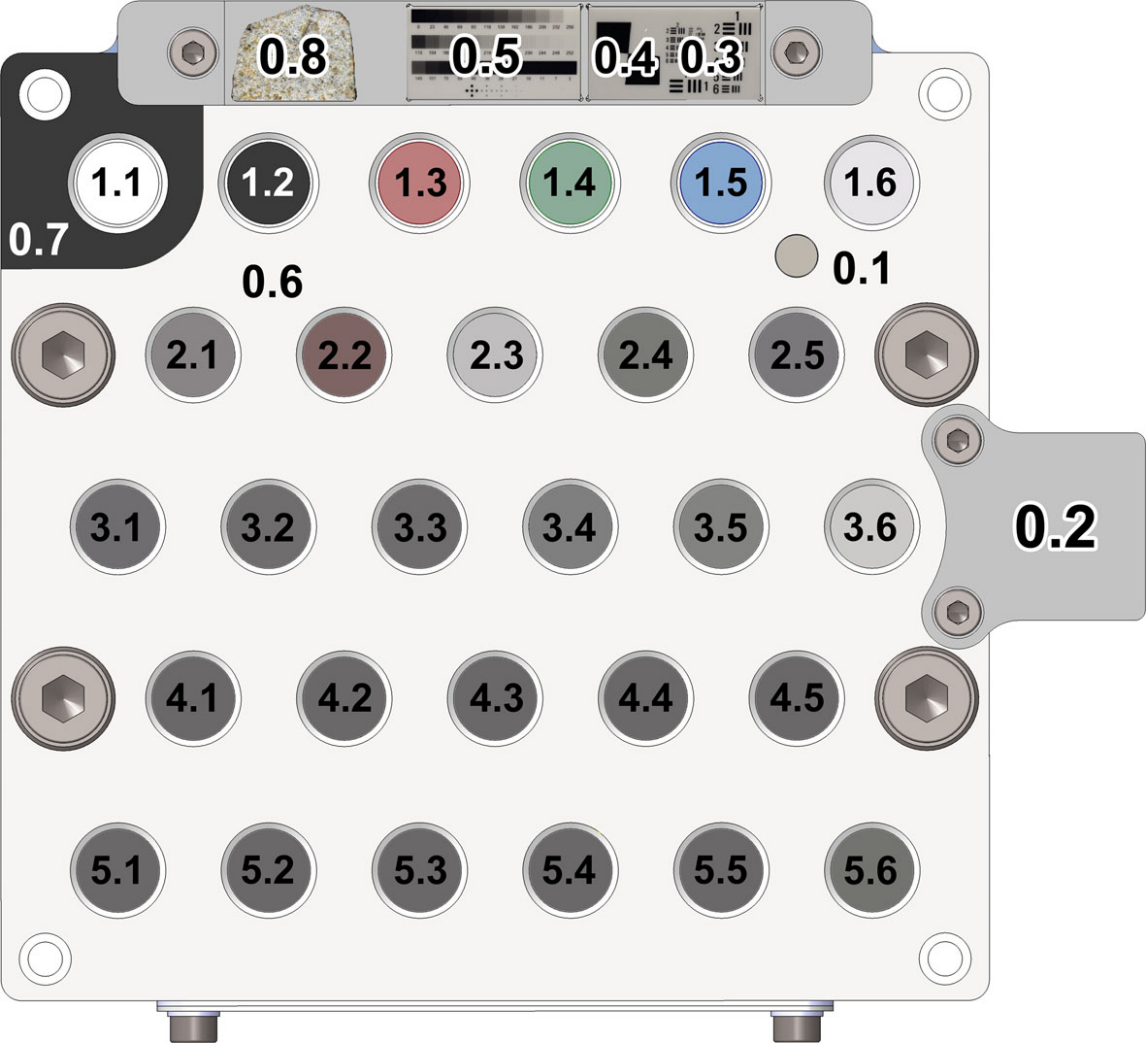
Arrangement of targets with identifiers for each of them

Each technique used by SuperCam has its own requirements in terms of calibration. In addition, cross calibrations, or correlations between different techniques, are needed for synergistic combination of data. It is key, for example, to understand from where in the field of view of the imager an infrared spectrum was taken, or to correlate chemometric calculations from LIBS with a certain mineral identified by Raman. To fulfill all these needs SuperCam relies on the SuperCam Calibration Target (SCCT) (Fig. 2 and Table 1), an assembly consisting of a collection of samples made to satisfy the above-mentioned calibration needs, and a mechanical assembly in which the targets are located. The whole system was designed to withstand the thermal and mechanical requirements of the mission.

**Table 1:**
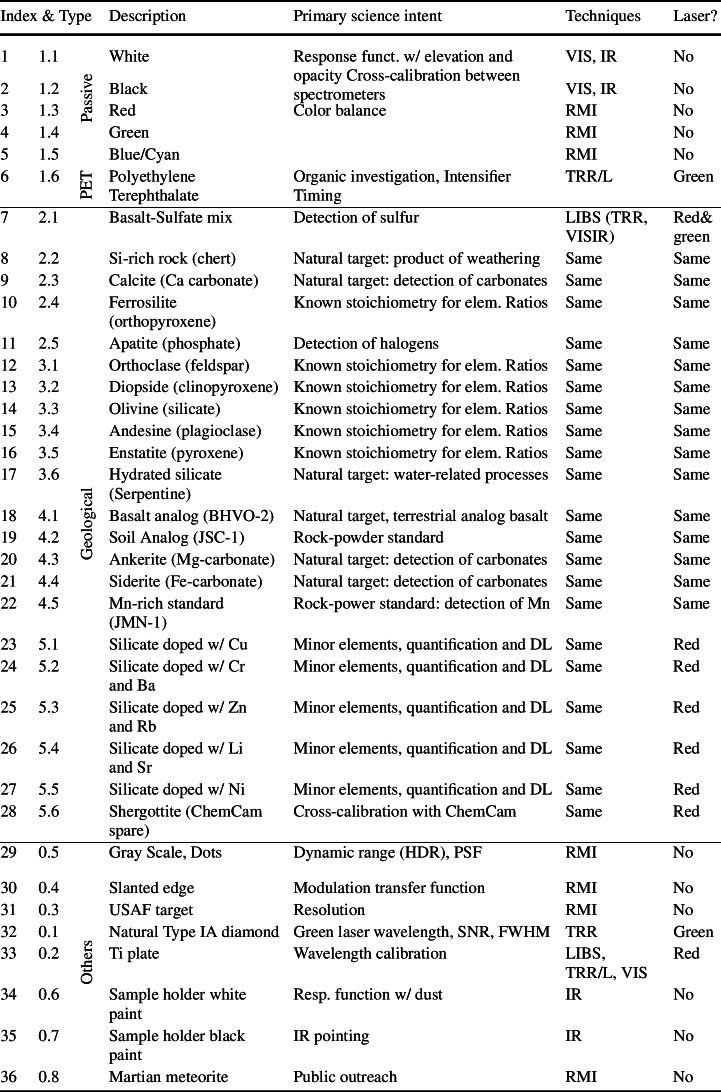
SuperCam calibration targets and rationale for their inclusion. Indices (2nd column) refer to Fig. 2

Notes. Acronyms are: high dynamic range (HDR), point spread function (PSF), signal-to-noise ratio (SNR), full width at half-maximum (FWHM), detection limit (DL)

This paper aims to describe the development of the SCCT. It has been organized in four main sections. The first one describes the targets, the scientific and technical requirements leading to their final selection and their characterization and science testing. The second section covers the holder development process. The third section describes the model philosophy and the validation process in the path to the final Flight Model (FM), now on board the Perseverance Mars 2020 rover. A final section summarizes the results obtained in terms of planetary protection and contamination control, both important since the SCCT is external to the rover.

## SuperCam Calibration-Target Description

Every scientific instrument needs calibration. Small motions in the hardware during cruise, or aging, can induce changes that, if not identified, can cause signal changes being misinterpreted. Evolution of the optical response function needs to be tracked, since it may impact the accuracy of the chemometric calculations done with LIBS data. Spectral accuracy is key for the identification of the different atomic lines with LIBS, but also with Raman spectroscopy. For instance, the position of the main band of some carbonates can vary by ∼1 nm. Infrared spectroscopy requires frequent re-calibration because of the changing characteristics of the atmosphere. These are just a few examples on how critical it is to have a reliable calibration of the instrument in situ.

SuperCam’s calibration began during the development of the instrument, at the Mast-Unit level (mostly for the RMI, IR, MIC, and the laser’s irradiance) or at the Body-Unit level (mostly the spectrometers’ characteristics). Additional tests have been carried out after the two parts were integrated on the rover, during the Assembly, Test, Launch, Operations (ATLO) activities at the Jet Propulsion Laboratory (JPL). Results are discussed in Maurice et al. (2020, this issue) and Wiens et al. (2020, this issue). Although the acquired dataset is generally sufficient for scientific use of the data, there are several reasons to extend the calibration activities on the surface of Mars:

- To perform a health check and performance status after the rigors of launch, cruise, entry-descent-landing, and mast deployment;
- To evaluate the actual performance of the instrument in a relevant operational environment where measurements are done. This allows the team to identify the effects of temperature, pressure, and dust;
- To track any performance drift with time due to component aging and dust accumulation;
- To provide a “ground truth” solution for SuperCam quantitative analysis based on spectral data from the different techniques; this is particularly important for LIBS spectra.

After every calibration acquisition done on the SCCT, Mars data will be compared to laboratory calibration data. These data will enable the development of transfer functions from measurements done in the Martian environment to Earth-based observations using instrument replicas, including laboratory calibration to Mars conditions (Clegg et al. 2017). To respond to these three overarching requirements, SuperCam carries a suite of 36 calibration targets that were carefully chosen and characterized. This section explains the scientific and technical drivers for the choice of each of them and their intended use; Table 1 summarizes the targets. Each of the 36 targets has a “X.X” code is this table and in Fig. 2.

Once the targets were installed, it became clear to the team that their purpose could be extended as follows:

- To derive instrument characteristics (focus, pointing) that could not be tested prior to launch due to lack of time, or new activities not foreseen before;
- To conduct science studies (speed of sound, aging of organics on Mars, atmospheric dust deposition).

This is also described hereafter. Finally, we should remember that the SCCTs are all at the same distance of ∼1.56 m from the Mast Unit, a distance that is not relevant to the actual distances of most targets (≥ 2 m). The scope of the calibration on Mars is necessarily incomplete and will need to be extended to several distances in the laboratory. The number of targets is also limited: many more calibrations (e.g., 400 + samples for LIBS) will also be performed in the laboratory. For these reasons, two high-fidelity models of SuperCam will complement the in situ calibration during the course of the mission, at LANL (Los Alamos, NM) and IRAP (Toulouse, France).

### RMI calibration: Geometric Target

**Fig. 3:**
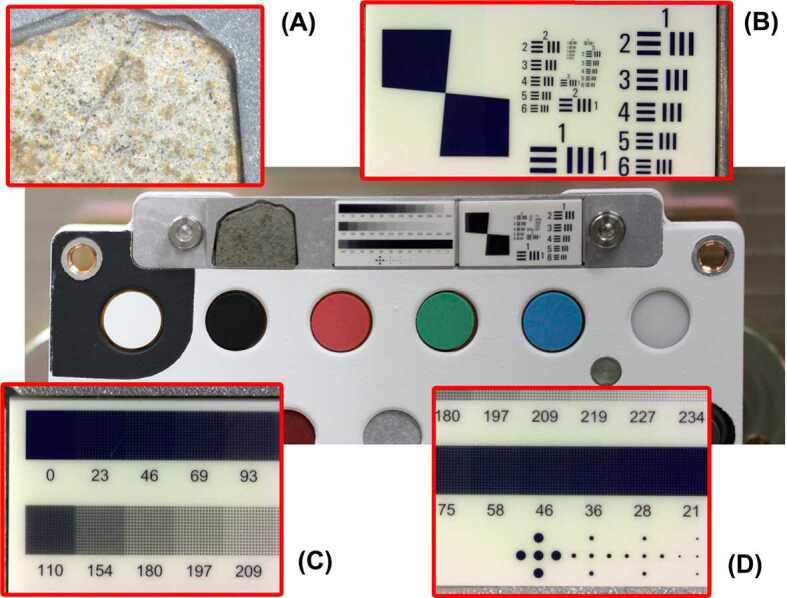
The geometric target provides two different patterns that help with different imaging calibrations. A gray scale is for white and HDR calibration (**c**), and different geometric patterns are shown (**b**, **d**) that can be used to fine adjust the autofocus and estimate the resolution of the system

The RMI performance was well characterized prior to launch as a function of distance and temperature (Maurice et al. 2020, this issue). Some key characteristics, such as the resolution of the system or the dynamic range of the camera, will be checked on Mars. Two targets to diagnose the resolution and the modulation transfer function (MTF) are provided on one small plate (detail B in Fig. 3); targets for dynamic performance and point spread function (PSF) diagnostics are on another one (details C and D in Fig. 3). Both plates are made of 99.6% alumina (aluminum oxide) covered with “patterns” consisting of anti-reflective chrome plating ($R < 10$% in the visible) with a spatial accuracy of ±0.25 microns. Plates are $16.5 \times 9.0 \times 1.27$ mm (±0.1). The center of the whole assembly, which also includes the Martian Meteorite (NWA 10170; detail A in Fig. 3), is located at 1.565 m from SuperCam’s Schmidt plate (aspheric lens in the Mast Unit, see Maurice et al. 2020, this issue). At this distance, the angular extent of each alumina plate is 10.5 mrad, about half of the RMI field of view (18.8 mrad). Two to four calibration images will be necessary to center each reference pattern in respective images. Targeting with the laser on these plates is prohibited, per SuperCam flight rules.

The optical resolution – the ability to distinguish objects by their separation – is determined using a target similar to a United States Air Force (USAF) resolution test chart. Each element is made of three identical closely spaced bars in the horizontal and vertical directions (detail B in Fig. 3). The resolution $x$ in line pairs/mm is given by $x = 2^{G + (E - 1)/6}$, where $G$ is the group number (from 1 to 4 in the case of this target) and $E$ is the element number within the group (from 1 to 6). In both directions, the requirement is 80 μrad for >20% contrast across the bars. At a distance of 1.565 m, that corresponds to 125 microns for a white-black pair, or 7.99 pairs/mm. Hence the requirement is met when a 20% contrast is found at group 3, element 1 ($G=3/E=1$). Simulations shows that we could expect to reach 9.13 line pairs/mm, or $G=3/E=3$. The best possible resolution for the Mast-Unit telescope design is $G=4/E=6$, which corresponds to 22 μrad, which is slightly greater than 2 pixels at the center of the RMI field of view. It is not expected to be able to resolve objects smaller than twice that size, thus corresponding to $G=4/E=1$.

The MTF can be determined directly from a high-contrast slanted edge at 5° (left part of detail B in Fig. 3) (Li et al. 2015; Masaoka et al. 2014, and references therein). In a nutshell, a one-dimensional line showing the intensity profile (also called the edge-spread function or ESF) is determined in the direction perpendicular to the edge. The derivative of the ESF is the line spread function (LSF) which is then “Fourier-transformed” into the MTF. The calculation is repeated in both vertical and horizontal directions.

The RMI CMOS (complementary metal oxide semiconductor) sensor (Maurice et al. 2020, this issue, Sect. D.3.2) has a shallow pixel well depth of 13.5 ke^−^ compared to 220 ke^−^ for ChemCam’s RMI CDD (charge coupled device). To cope with this limitation and to regain some dynamics, two high dynamic range (HDR) modes have been implemented (Maurice et al. 2020, this issue, Sect. D.9.2). To test them on Mars, 3 bands of 12 levels of grey are used (detail C in Fig. 3). The different tints are built from various densities of $5 \times 5$ μm chrome-plated pixels. They are grouped as patterns of 16 by 16, which are printed in spirals as the density increases. It uses 257 levels from black (index = 0) to white (256). The upper row is linear from 0 to 256. The second and third rows are logarithmic from 110 to 252 for light tones and from 145 to 3 for dark tones, respectively.

The PSF is an important characteristic of the RMI if deconvolution is to be used to sharpen the images. It can be measured from dot patterns whose size corresponds to the resolution of the system. Hence we have designed dots with diameters of 266, 125, 110, and 64 μm (detail D in Fig. 3). The size of these points corresponds respectively to 170 μrad (resolution at the border of the field of view), 80 μrad (required resolution at the center), 70 μrad (simulated resolution), and 41 μrad (optimal resolution), all of them at the distance of the SCCT (1.56 meters). The same target will be used to check the image distortion that is known to be $\leq 0.5$% over 10 mrad in diameter, and $\leq 2.5$% over the RMI field of view, 19 mrad.

These targets are somewhat sensitive to friction, and special care has been taken not to wipe them during cleaning. A small number of streaks or small blemishes are visible only at high magnification and will therefore be barely visible with the RMI and will not affect the analysis. During a quality-control inspection, however, it was noticed that the contrast between the dark lines and the white background was not as sharp as the engraving precision, which may be due to the optical limitations of the control system. If true, this may restrict the resolution analysis to an upper limit (greater than the 160 microns at 2 meters given as the resolution limit, Maurice et al. 2020, this issue). Finally, as soon as the rover lands, dust deposition will slowly degrade the quality of these targets. Dust deposition will be somewhat attenuated by the target’s tilt ($\sim 60$ deg from the rover deck) and occasional cleaning wind episodes like those observed by previous rovers. On the other hand, this dust accumulation can be used to study atmospheric deposition rates (Kinch et al. 2015).

Regarding the mounting of the geometric targets, an additional tilt for the two plates has been introduced with regards to the telescope boresight. Hence the plates are perpendicular as possible to the instrument’s boresight. Distortions will be taken into account for calibrations. In the case of the azimuth, this maximum angle is 6°, meaning that effects on the apparent size of the objects projected in the plane of the RMI could be negligible, under a 1%.

### Martian Meteorite

As a nod to the notion of return samples that this mission prefigures, and for outreach purposes, we have installed a ${\sim} 11 \times 9 \times 1.25$ mm slab from a Martian meteorite (ref. North West Africa, NWA 10170) in the mounting bracket of the RMI geometric target (Fig. 3m detail A). This Martian meteorite is mounted in a no-laser region of the holder (per SuperCam flight rules) and is not intended for LIBS or Raman analyses, but could be interrogated by the IR spectrometer.

**Fig. 4:**
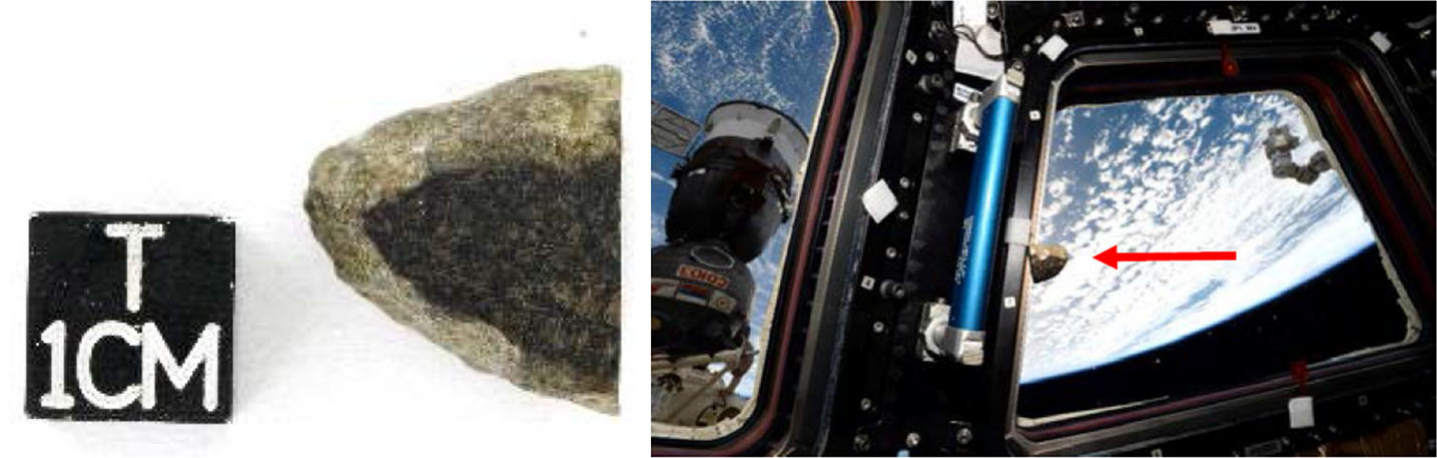
Left, the SuperCam NWA 10170 (Shergottite) meteorite placed on the geometric target before it was sliced (a 5.65 g fragment). Right, the meteorite floating inside the International Space Station (ISS). Credit ESA/T. Pesquet

This meteorite was found near the Moroccan-Algerian border and was purchased by L. Labenne at the Tucson salon from an anonymous dealer (Fig. 4) for the Toulouse Science Museum. The meteorite was then carried by the French astronaut T. Pesquet to the International Space Station, during his stay from November 2016 to June 2017 (Fig. 4, right). After his return, the meteorite was cut to be implemented in the SCCT. Once it lands on Mars, the meteorite will have passed through the Martian atmosphere twice and Earth’s four times.

Its composition, studied by R. Hewins and B. Zanda at the Museum National d’Histoire Naturelle de Paris (Bouvier et al. 2017), reveals olivine and pyroxene, with olivine phenocrysts from 100 μm to 1.5 mm in size. These crystals are embedded in a mesostasis of prismatic to subophitic pyroxene in a feldspar matrix (detail A in Fig. 3). This feldspar is presumed to be maskelynite, while the olivine composition is Fo_67.2 ± 7.7_ (Fa_20−42_), with a FeO/MnO ratio of 52.1. The pigeonite has an average core composition of En_65.8 ± 1.5_ and the atomic Fe/Mn ratio is 31.9. Its Fe/Mn ratio is characteristic of Martian meteorites, and it is classified as an olivine-phyric Shergottite.

### Passive Targets

Samples 1.1 to 1.5 in Fig. 2 and Table 1 are the reflectance standards used for radiometric calibration of the RMI and the VIS and Infrared Spectrometers. Reflectance standards are described below in the order they appear in Fig. 2:

#### Sample 1.1 - White Sample

this target is used for the white balancing of images obtained by the RMI and characterization of the incident spectrum of the Sun. There needs to be a white reflectance standard with a behavior as close to Lambertian as possible (to avoid specular reflections) and resistant to UV radiation. The AluWhite98 material provided by Avian Technologies (New London, NH) was selected for this purpose.

#### Sample 1.2 - Black Sample

besides serving as a black standard for imaging calibration, the aim of this target is to characterize the dark current of the Infrared spectrometer. To fulfill this requirement, the sample needs to be black not only in the visible wavelength range but also as dark as possible in the infrared wavelength range to 2600 nm, which is the range used for the reflectance measurements by the IR spectrometer (Maurice et al. 2020, this issue). The sample consists of a disk of aluminum painted with Aeroglaze Z307, a polyurethane-based black paint exhibiting extremely low reflectance throughout the desired range of wavelengths (Beck et al. 2012) and having relatively high resistance against mechanical wear, and wide space usage.

#### Samples 1.3 – 1.5 – Samples of Primary Colors, Red, Green and Blue (RGB)

a set of red, green and blue/cyan reflectance standards produced by Lucideon (Staffordshire, UK) was selected. These samples are made of a fused clay with matted surfaces of three different color glazes.

**Fig. 5:**
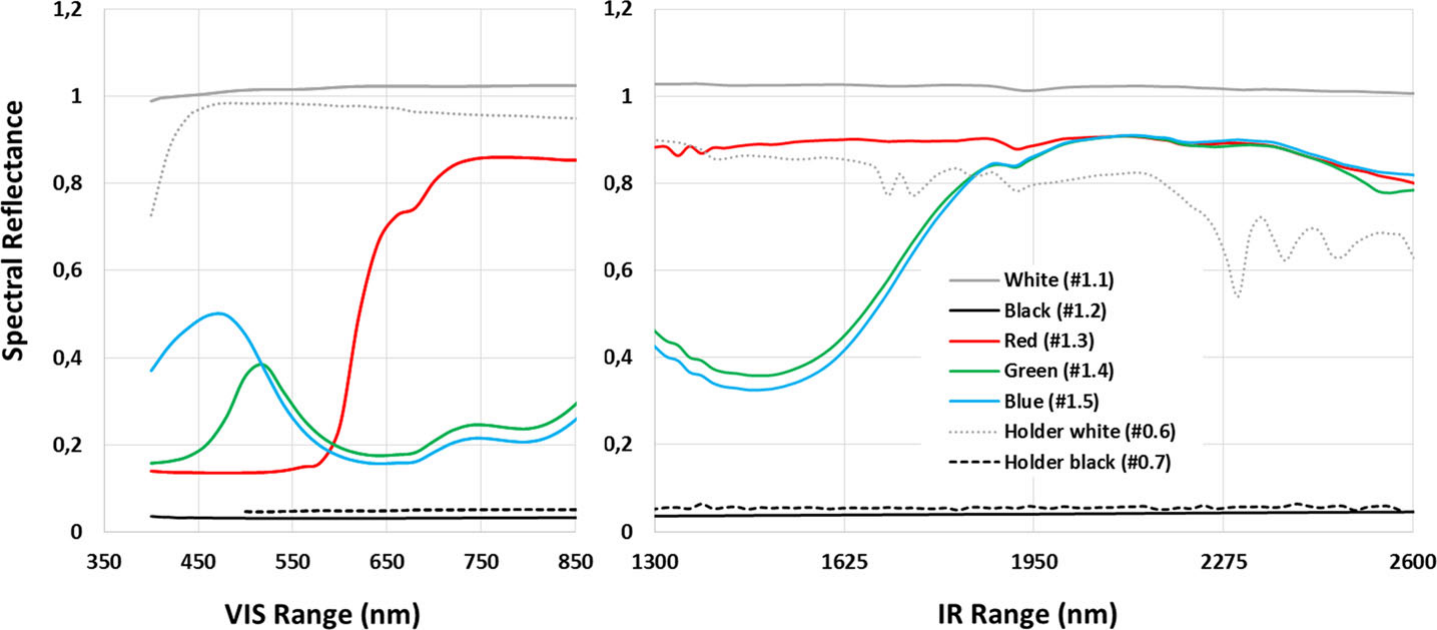
Spectral reflectance of passive targets for the VIS (400 – 853 nm) and IR (1.3 – 2.6 μm) ranges

The photometry of all reflectance standards has been characterized using a suitable spectrogoniometer setup (Beck et al. 2017; Buz et al. 2019) in Grenoble, France. Spectral reflectance is shown in Fig. 5 for the different passive targets, with the incidence angle at 0° and an exit angle of 30°.

### Organic Sample

The SCCT carries a very stable organic-rich target made of polyethylene terephthalate (PET, target #1.6 in Fig. 2 and Table 1). The purpose of this target is to ensure that SuperCam can detect organic molecules on Mars. Thanks to the signal intensity, the PET target can also be conveniently used by the engineering team to verify the timing between the start of the integration window and the laser pulse: synchronization will be checked using time-sweep experiments, i.e. by varying the delay of the intensifier gate until the best value is obtained (Wiens et al. 2020, this issue).

The chemical composition of this target, including aromatic, aliphatic and ester/carboxylic functional groups, is representative of expected organic compounds trapped in ancient rocks (Bernard and Papineau 2014). The science team intends to use this target to perform aging experiments under Mars-surface UV radiation. Early studies at the Astrobiology Center in Madrid, using the Planetary Atmospheric Surface Chamber (Mateo-Martí et al. 2006; Mateo-Marti 2014) and a UV dose equivalent to 230 sols, showed that aging of PET can be detected as a signal-to-noise reduction and a change of the band profile (Lopez-Reyes et al. 2020). The presence of perchlorates can alter this process or induce chemical changes that could be observed as changes in its Raman spectral signature (Góbi et al. 2017). Therefore, the target will be measured by SuperCam regularly during the mission.

This material was also selected as a calibration target for the Raman Laser Spectrometer (RLS) onboard the ExoMars Rover (Lopez-Reyes et al. 2020; Rull et al. 2017). In contrast to that of ExoMars, this PET target is 100% crystalline and was machined at IMPMC in Paris out of an Ertalyte^®^ rod provided by Mitsubishi Chemical Advanced Materials. The surface roughness was reduced through gentle polishing, keeping the same size as the other targets (1 cm diameter, 5 mm thickness).

PET is a thermoplastic polymer (C_10_H_8_O_4_)_n_ known for its excellent combination of mechanical and thermal properties, as well as its chemical resistance (Barber 2017). Thanks to its high crystallinity, the melting point of this target is above 250 °C while its boiling point is > 350 °C, which means that it does not suffer plastic deformation during the Dry Heat Microbial Reduction (DHMR). This is a Planetary Protection measure required for the SCCT, during which the material is exposed to temperatures higher than 110 °C for several hours, reducing the population of spores.

**Fig. 6:**
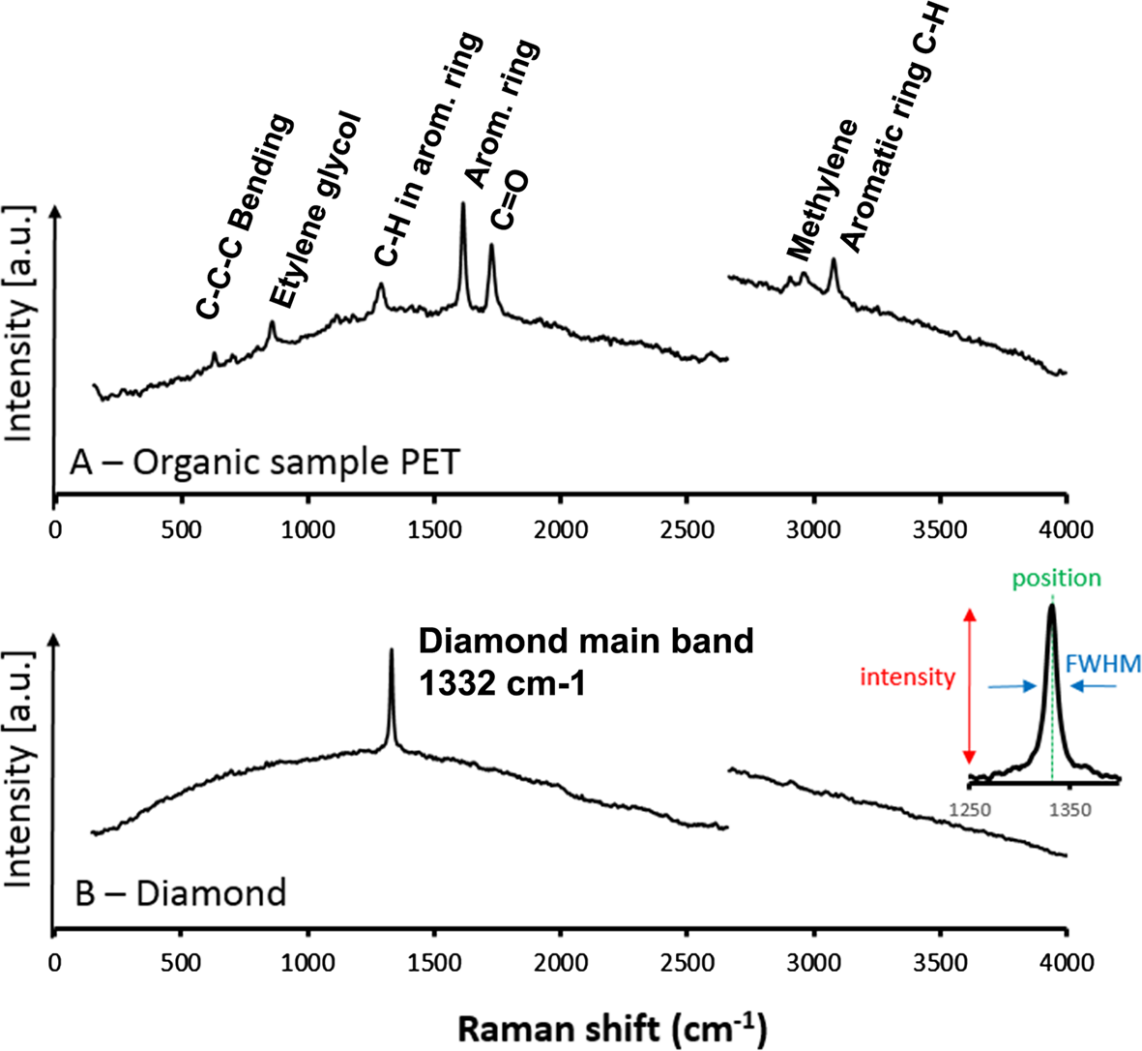
Raw spectra acquired by SuperCam during ATLO tests at JPL. (**A**) Raman spectrum of the organic PET target. Spectrum acquired by SuperCam using 10 single shots. See text for the peak identification. (**B**) Raman spectrum of the Diamond showing its main band at 1332 cm^−1^. The luminescence background from the adhesive under the diamond piece can be seen. The main parameters used for checking the instrument health are depicted in the right inset

The Raman signal of PET is usually very strong (Rebollar et al. 2014). A high signal-to-noise ratio is obtained with just a few laser shots, as verified during ATLO tests at JPL, from a series of 10 shots and an integration gate of 100 ns (Fig. 6A). Key features are numerous and readily identifiable: 633 cm^−1^ (C−C−C in plane bending), 859 cm^−1^ (left ethylene glycol), 1095 cm^−1^ (C−C bonds), 1120 cm^−1^ (ester C(O)−O), 1288 cm^−1^ (ring C−H bending in-plane), 1612 cm^−1^ (ring mode), 1726 cm^−1^ (stretching C=O), 2966 cm^−1^ (methylene groups adjacent to oxygen atoms), 3083 cm^−1^ (aromatic C−H bond).

### Diamond

Diamond is commonly used in laboratories as a rapid, easy-to-use calibration target for Raman instruments, as it provides one of the strongest well-known Raman signals for solid materials. Due to the high point-group symmetry of its crystal structure and its high purity, diamond shows only one first-order Raman band at ∼1,332 cm^−1^, which has a narrow FWHM < 2 cm^−1^ (Krishnan 1945; Solin and Ramdas 1970). This band presents no shifts or variations due to temperature or pressure in the range to be experienced by the SCCT, another important factor considered in its selection as a calibration reference (Fig. 6B).

SuperCam’s diamond (target #0.1 in Fig. 2 and Table 1) is a circular slice, 4 mm in diameter and 1 mm in thickness, which was cut from a monocrystalline sample. This diamond is a natural type IA and has been provided by Almax easyLab, Belgium, under the reference number Almax easyLab P01641. Such type IA natural diamond contains a trace amount of nitrogen (∼2.1 ppm) and exhibits a low-fluorescence background. The slice was laser cut from the raw crystal in the (100) orientation, chamfered on one side and polished on both sides. The homogeneity of the signal from diamond target was verified by performing Raman hyperspectral mapping to check the uniformity in peak position (<1 cm^−1^), and FWHM (<0.5 cm^−1^) over its entire surface.

This Raman band is so intense that it can be easily detected with a single laser shot by SuperCam, and summing tens of laser shots will yield an excellent signal with good statistics. A variation of the position of this band would directly inform the team about a potential issue with wavelength calibration, which is obtained in an absolute sense from LIBS spectra from the Ti plate (see below). Since the wavelength of a Raman band is relative to the excitation wavelength, if the wavelength of this Raman band is known accurately, it can also be used to assess the wavelength of the laser. The green laser beam was calibrated in wavelength early (Maurice et al. 2020, this issue). It varies linearly between 531.88 nm (Mast Unit at −40 °C) and 532.03 nm (20 °C; both terrestrial air-pressure wavelengths), which translates to a shift in Raman bands within a range of 5 cm^−1^. The measurement of the wavenumber of the diamond’s Raman band allows us to check this shift.

The diamond band intensity will be used to verify the alignment (e.g., between the incident laser beam and the telescope field of view). Because the FWHM of the measured diamond band is actually sharp and is significantly lower than the instrument’s spectral resolution (9-12 cm^−1^), the measured FWHM of the diamond target will therefore be used to monitor the instrument’s spectral resolution.

The diamond’s size (Ø 4 mm, 2.56 mrad) is smaller than other targets, but significantly larger than the TRR/L field of view (0.7 mrad). The mast pointing at the center of the diamond will be challenging, and several attempts to center the laser beam may be necessary early in the mission. The diamond is bonded by an epoxy paste adhesive (Henkel/Loctite EA-9309NA) that generates a fluorescence background. Therefore, a lower intensifier gain should be used, compared to the other targets, to avoid saturating the CCDs in the Body-Unit spectrometers due to this background.

### Titanium Plate

A baseline wavelength calibration has been determined before launch for the Body-Unit spectrometers. These spectrometers are very stable with temperature, much more than ChemCam’s spectrometers (Wiens et al. 2020, this issue). However, there are residual wavelength shifts, typically 1 pm/°C and a maximum of 3 pm/°C (Wiens et al. 2020, this issue). This means that the position of spectral lines will move by ∼0.035 nm to 0.105 nm over the 0 °C – 35 °C range of temperatures considered for the Body Unit on Mars. Considering the pixel resolution, 0.048 nm/pixel for the UV spectrometer (245-340 nm), 0.042 nm/pixel for the VIO spectrometer (385-465 nm), and 0.064-0.090 nm/pixel for the TSPEC (536-853 nm), such drifts correspond to 1-2 pixels. In terms of resolution in wavelength (0.12 nm FWHM for the UV and VIO spectrometers, 0.35 nm FWHM for the TSPEC), this is significant. Taking into account the possibility of long-term drift, which has been observed on ChemCam, it becomes obvious that a means of wavelength recalibration is necessary. To that end, the SCCT carries a Ti6Al4V alloy target (19 mm x 18 mm), also called the “Ti plate” (Target #0.2).

A LIBS spectrum of titanium has >300 major emission lines between 245 nm and 850 nm, the wavelength range of interest to SuperCam. This density of intense lines makes it a very recognizable spectrum, the features of which are adapted for pattern recognition to recalibrate the wavelength of spectra taken under different conditions. The algorithm for wavelength recalibration will be the same matched filter technique that was developed for ChemCam (Clegg et al. 2017; Wiens et al. 2013). The accuracy of this technique is better than 0.2 pixel (e.g., to < 10 pm for some spectral ranges), which is the accuracy required to apply multivariate technique analysis in quantifying the LIBS spectra and to provide high-precision wavenumber calibration for Raman spectra. The correction in wavelength will be referenced to the baseline calibration obtained in the laboratory based on known vacuum emission lines. Mars atmospheric pressure, at ∼1% of the standard terrestrial pressure, is close to vacuum conditions and well within the wavelength uncertainty (Clegg et al. 2017).

**Fig. 7:**
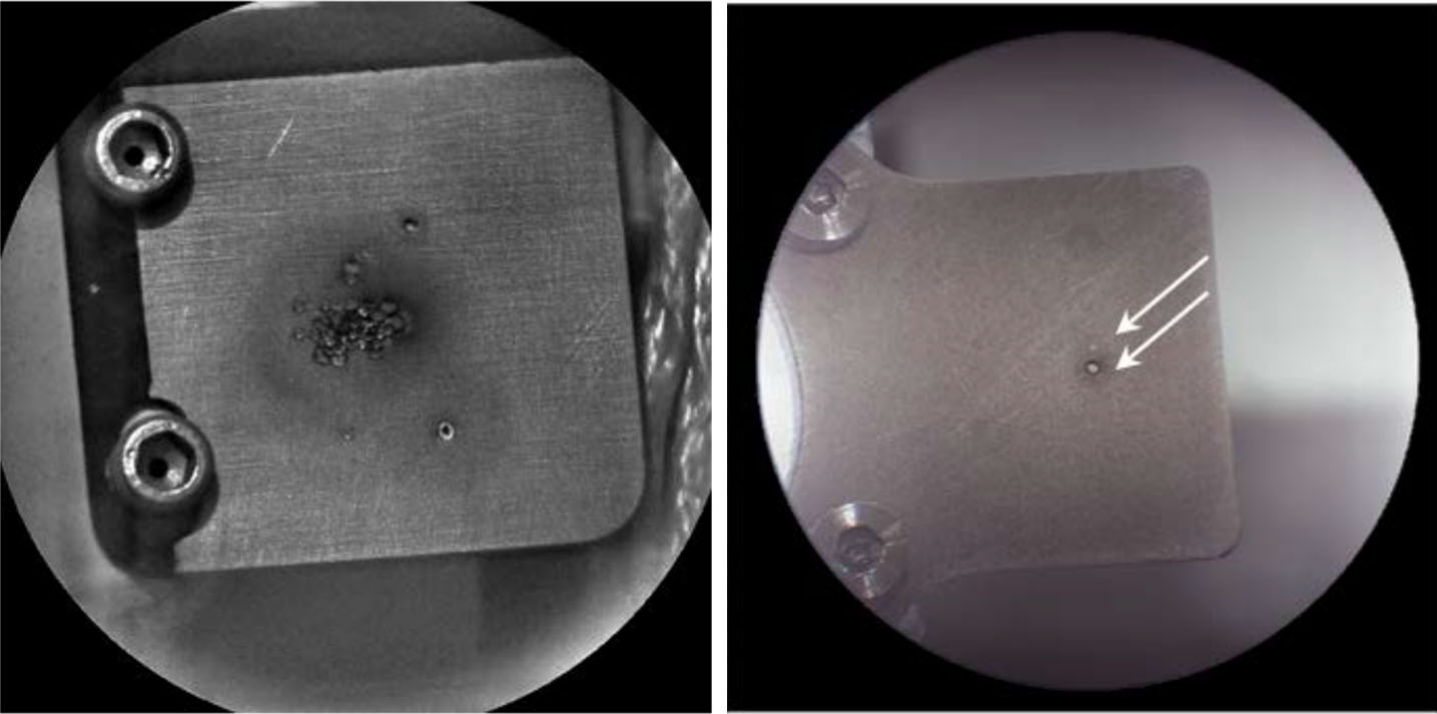
(Left) RMI image of ChemCam Ti plate on board MSL on Sol 2276, (12/31/2018). (Right) RMI image SuperCam Ti plate after two bursts (arrows) during ATLO tests at JPL

Titanium LIBS observations will be made over a range of spectrometer temperatures with a frequency that depends on how the spectrometers perform. The laser energy and/or the number of detector integration rows will be reduced so as not to saturate the spectra (Wiens et al. 2020, this issue). As of this writing on MSL Sol 2738, ChemCam has shot the Ti plate 6,000 times (bursts of ∼30 shots). That corresponds to one burst every 2 weeks. In Fig. 7 (left) a ChemCam RMI image of the Ti plate on Sol 2276 is depicted. As shown, prior to launch, SuperCam’s Ti plate (Fig. 7, right) has been shot only twice during ATLO tests at JPL: once during the thermal tests at cold (30 shots, faint spot shown by the top arrow), and once at room temperature (35 shots, large pit shown by the lower arrow). The size of the pit depends on the quality of the focus. (As a reminder, ChemCam’s RMI is a monochrome imager, contrary to SuperCam’s.)

According to this plan, LIBS of the Ti plate will provide data for temperature-dependent channel-to-wavelength conversion laws for all Body-Unit spectrometers. The TRR/L wavenumber calibration will be obtained at the same time, and can be checked or corrected for other effects using the diamond target; see previous sections.

Finally the Ti plate will be used to determine the speed of sound on Mars, thanks to the capability of SuperCam’s microphone to record the LIBS-induced shock wave (Chide et al. 2020, 2019).

### Geological Targets

The objectives of the LIBS targets are 1) to detect any performance drifts with time; 2) to compare Mars data with laboratory spectra of the same targets (transfer function between the two environments); 3) to understand in situ the effects of various instrument parameters on the spectra collected, such as laser energy, focus quality, or spectrometer gains; 4) to be a subset of a larger database (400+ targets) that is used in the laboratory to obtain quantitative elemental composition (Clegg et al. 2017; Wiens et al. 2013); and 5) to compare with ChemCam results, sharing duplicates of the same target. Overall, LIBS requires a library of standards to obtain quantitative compositions via calibration curves or more complex multivariate techniques to untangle matrix effects (Clegg et al. 2017; Fabre et al. 2011). The library must include targets of similar mineralogy and elemental compositions to the field targets of interest. Having mineralogical end-members and analog targets on board assures the best opportunity of high-accuracy calibration.

Table 1 summarizes all the different targets included in the FM SCCT along with their science rationale. With the exception of the chert sample and the shergottite from ChemCam, most of the LIBS targets were manufactured in different laboratories in France under IRAP’s coordination. The manufacturing included the selection of a good natural candidate, crushing it into a fine powder and its later compression into a pellet using flash sintering (Biesuz and Sglavo 2019). This process allows the manufacturing of pellets that are more homogeneous in both mineral and elemental distribution than the original rock. In some cases, this process can imply a change in mineralogy due to the occurrence of phase transitions. However, as the samples have been largely characterized after SPS and before launch, and this does not affect the Raman calibration. The chemistry is not affected by such mineralogical changes (Montagnac et al. 2018; Manière et al. 2019), in addition they have been characterized for their chemistry as well.

The process of sample selection started before the selection of Jezero as landing site. Nevertheless, orbiter data from the candidate sites was taken into account in the selection of the geological samples of the SCCT. Jezero Crater provides access to a wide repository of poorly-altered igneous rocks (Liu et al. 2019). Several samples, from 2.4 to 3.5 in Table 1 and Fig. 2, can be used to calibrate their chemical composition, from which important insights about the evolution of the crust-mantle system could be inferred (Grott et al. 2013).

The carbonate-bearing units detected along the inner crater rim have been related to possible fluvial-lacustrine activities (Horgan et al. 2020). SCCT samples 2.3, 4.3, 4.4 and 4.5 can be used to aid in LIBS and Raman discrimination among carbonate phases, whose mineralogical composition should fall within the ternary system CaCO_3_ - MgCO_3_ - FeCO_3_ (Horgan et al. 2020).

Characteristic spectral features of serpentine were also detected in Jezero crater (Noe Dobrea and Clark 2019), thus confirming the past occurrence of water-alteration processes. The lamellar structure of this mineral could have retained and preserved biomarkers. It may therefore represent an optimal scientific target for the potential detection of life tracers on Mars, being a priority mineral for the objectives of Mars-2020 mission (Fornaro et al. 2018). Because of this, serpentine was included in the SCCT as sample 3.6.

In addition, doped glasses (sample batch from #5.1 to #5.5) will be used to calibrate the minor and trace elements that were found to be ubiquitous constituents of Martian rocks and soils (Payré et al. 2017). Similarly, the apatite reference sample 2.5 will be used to calibrate the halogen elements, whose study provides insight about many magmatic- and aqueous-related processes that have occurred on Mars (Rampe et al. 2018).

Some interesting minerals were not included in the SCCT. Clay targets (due to intrinsic material properties) could not be effectively processed in accordance to mechanical requirements (Montagnac et al. 2018). Besides sintering, thermal processes that needed to be performed on the SCCT and described in Sect. 4, prevented the inclusion of phyllosilicates or highly-hydrated minerals such as gypsum. However, from the LIBS point of view, the compositions covered by the samples included in the SCCT are spread a large enough range to characterize these minerals. In addition to this, an extended mineral library will be used with Earth-based instruments, including these particular samples.

To serve their calibration purpose, the geochemical and mineralogical composition of the selected samples needs to be well characterized and, at the same time, they need to be homogeneous in terms of their element’s distribution at a scale down to 100 μm (measuring spot size). These requirements were addressed in a complete set of analytical processes (Gómez-Nubla et al. 2018), that included:

- XRF (X-Ray Fluorescence) mapping for the elemental spatial distribution. Obtaining the elemental distribution to a scale down to 100 microns.
- Raman spectroscopy for the mineralogical variation.
- LIBS for a more sensitive comparison to our needs with SuperCam. Done in IRAP and Malaga University.
- Laser Ablation Inductively coupled plasma mass spectrometry (LA-ICP-MS) and Electronic Micro Probe Analyzer (EMPA), to characterize the composition of the samples.

The homogeneity analyses and results are part of a dedicated paper by J.M. Madariaga (in preparation), while the results from the characterization of the samples, along with their compositions are included in a separated paper by Cousin (in preparation).

### Dust Mitigation on the Targets

The reddish color of the Martian sky is caused by the presence of small airborne dust particles. This dust can harm different systems of the Rover and is one of the factors to be better understood before the human exploration of Mars. In the case of SuperCam and its Calibration Target, the possible impact of the dust comes from its possible accumulation on the surface of critical samples. This could make it difficult to do measurements on them due to the opacity of the dust.

In general, the tilted position of the SCCT (see the following Sect. 3) reduces the deposition of dust compared to flat surfaces, as could be observed on Curiosity’s deck (e.g., Vicente-Retortillo et al. 2018; Lasue et al. 2018). Various additional approaches to dust mitigation have been taken in different parts of the SCCT, as not all the targets are equally sensitive to the presence of dust. In the case of the geological samples or Ti plate, thanks to the shock wave generated during the LIBS plasma events that displace the dust from the surroundings of the impact point, dust is not expected to be an issue for LIBS. Because LIBS is done before Raman or VISIR measurements, the operations using these samples are expected to be much less affected by dust.

The situation is different for the diamond and the organic samples, which are not planned to be targeted by LIBS. The diamond sample has been mounted flush with the surface of the holder, preventing any lip where the dust could accumulate. The tilted position of the SCCT and the proximity of this target to two geological samples (cleaned by LIBS) are expected to minimize dust impact. In the case of the organic sample, given the purpose of this element, interaction with the dust is desirable, as could help us understanding if dust has possible impact in the ageing of the organics on Mars.

In the case of the targets used for imaging and VISIR (passive targets and geometric target), none of them are shot by the laser during operations, or are close enough to a LIBS target to benefit from the cleansing shockwave of LIBS measurements. In the case of the geometric target, as was mentioned before, an additional tilting of the plates ensures that the deposition of dust will be minimized during the operational life of the SCCT. The case of the passive targets is slightly different, as their reflectance spectrum is more sensitive to dust deposition than the geometric target, especially in the visible wavelengths. A different approach was selected to keep these targets clear of dust, described below.

The Perseverance Rover’s Mastcam-Z instrument is using a radiometric calibration target with eight ring-shaped permanent magnets (Kinch et al. 2020, this issue). The exact same technique is used for SuperCam’s reflectance standards (targets #1.1 to 1.5). In addition, the tilted position of the SuperCam calibration target provides some extra protection against airborne dust accumulation compared to the horizontal surface of the Mastcam-Z calibration target.

During the Viking missions to Mars it was discovered that a bull’s-eye shaped permanent magnet embedded in the reference test chart (RTC) on the landers attracted airborne dust already during the landing and that the amount of dust on this target increased during the course of the mission (Hargraves et al. 1979 and references herein). To learn more about the properties of the airborne dust a set of magnetic-properties experiments were included on NASA’s Mars Pathfinder lander (Gunnlaugsson et al. 1998). Results corroborated the earlier results from the Viking mission that the airborne particles did possess a significant magnetic susceptibility, causing them to be attracted even to magnets with a relatively weak magnetic field gradient. This result showed that some of the airborne particles are rather strongly magnetic (Madsen et al. 1999). One question remained after the magnetic-properties experiments on Mars Pathfinder, regarding the relative abundance of magnetic particles among the airborne dust in the Martian atmosphere. An attempt to answer this question was made by the use of a very strong sweep magnet onboard Spirit and Opportunity (Madsen et al. 2003). Results from imaging this small device by Pancam showed that a major fraction of the airborne particles in the Martian atmosphere is magnetic to some degree. This resulted in a small area in the center of the sweep magnet remaining visually clean throughout the mission (Kinch et al. 2015; Madsen et al. 2009). Based on this result, permanent magnets were embedded in the Mastcam radiometric calibration target on NASA’s Curiosity Rover (Bell et al. 2017).

**Fig. 8:**
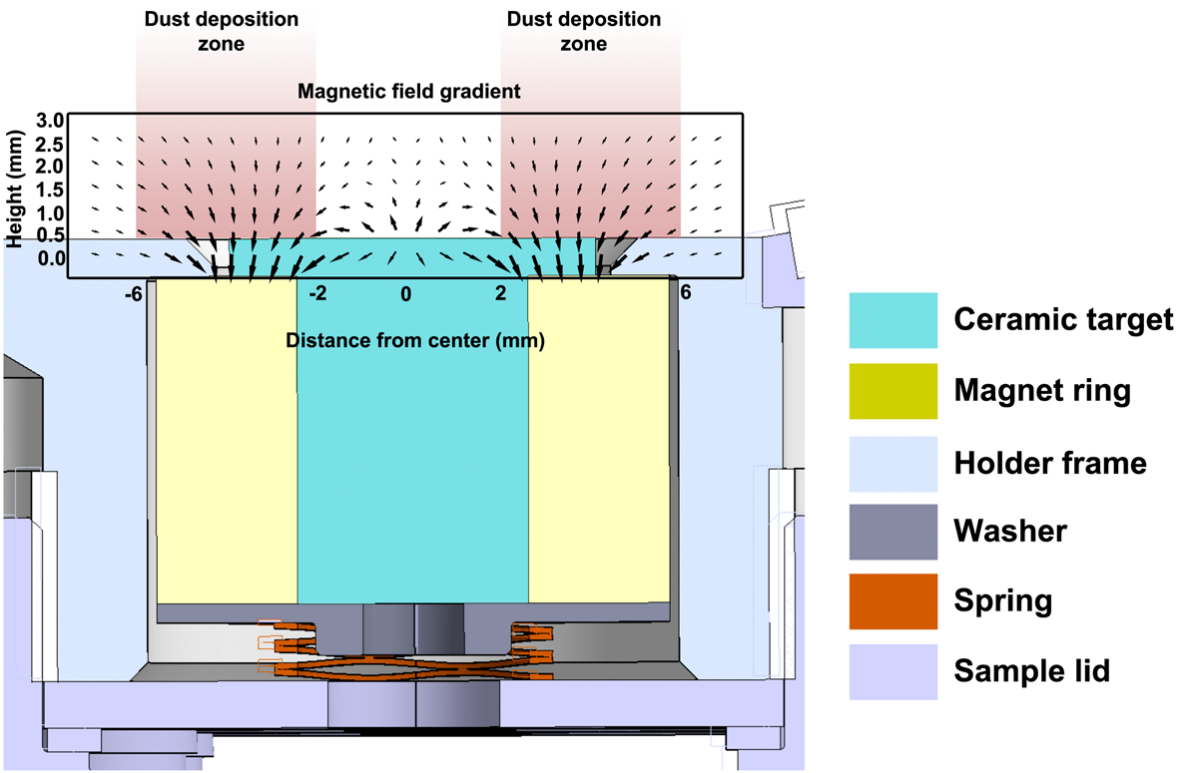
Passive sample cross section, where the magnet (yellow) can be identified wrapping the lower part of the reflectance standard (cyan), mounted on the SCCT holder (light blue). Magnetic field is represented defining the zones of deposition of the fraction of airborne dust with a significant magnetic susceptibility

**Fig. 9:**
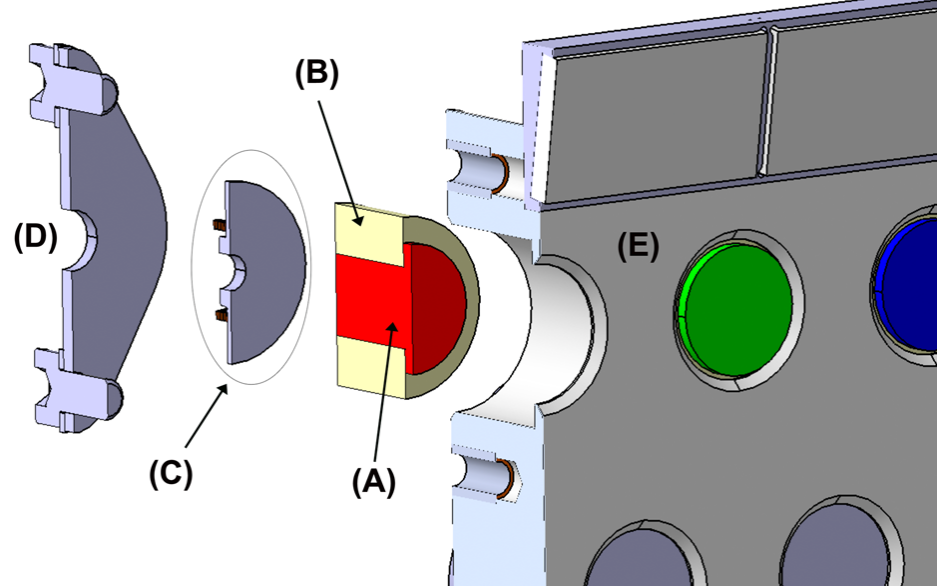
Cut-away view of how a passive sample is integrated into the holder. (**A**) Passive sample, (**B**) magnet, (**C**) protective washer, (**D**) closeout lid, (**E**) sample holder. Kapton^®^ shims are included between the sample and the protective washer, and also on top of the sample, covering only the magnet, and between this element and the holder. Finally, Kapton^®^ pieces wrap the whole target covering the lateral surface of the magnet

For SuperCam and Mastcam-Z, the circular reflectance standards have a T-shaped vertical cross section and are each glued into a Sm_2_Co_17_ ring-shaped permanent magnet (Figs. 8 and 9) that will keep the central ∼4 mm diameter of each target clear of airborne dust. Permanent magnets of the same design were used onboard the Phoenix mission (Drube et al. 2010). The magnets are produced from a delicate Sm_2_Co_17_ sintered alloy. The survival of these brittle components to mechanical stress during launch and pyrotechnic shock during landing is ensured by the structural resistance provided by the mounting system, described later.

## Mechanical Design of the Holder and Mounting on the Rover

The sample holder contains and protects the targets described in Sect. 2. It was designed by Added Value Solutions (AVS, Elgóibar, Spain). Among all the different requirements that needed to be taken into account, the most critical were:

- Mass budget: The requirement established by JPL’s payload office was to be under 270 g.
- From the mechanical point of view, most calibration targets are ceramic-like materials, therefore they are potentially brittle. This was a concerning issue, as the sample holder is situated in a high-g shock zone (3,500 g) of the rover. A conservative approach in the design was to plan in advance for a variety of target mechanical properties.
- The thermal range is very broad. To be compliant with planetary protection requirements, the SCCT had to pass DHMR tests at 115 °C. On the other hand, the targets must survive extreme Martian cold temperature, −135 °C.

**Fig. 10:**
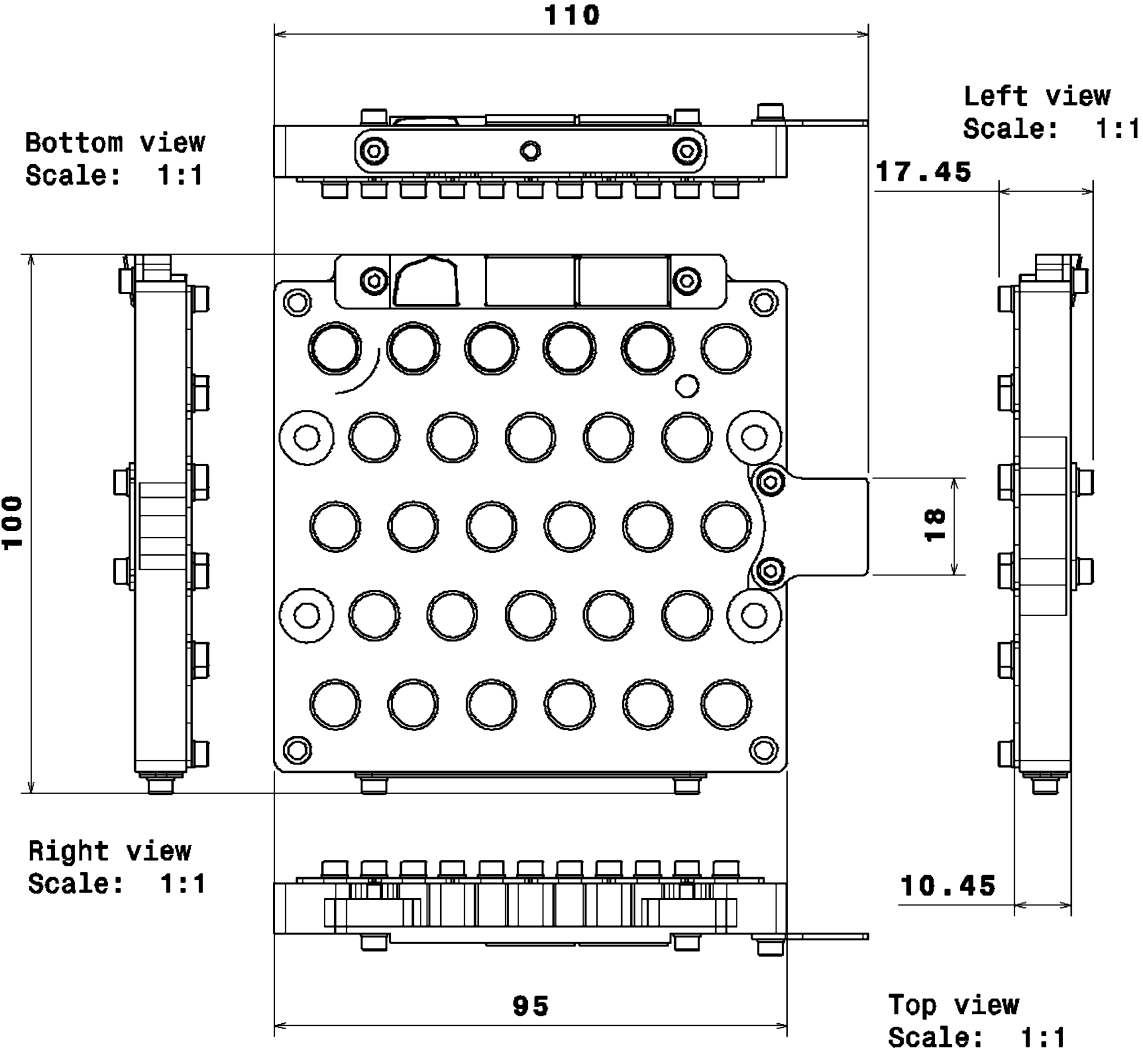
General dimensions of the SCCT. The total mass of the whole system is 248.5 g, with targets. See Fig. 2 and Table 1 for targets information

**Fig. 11:**
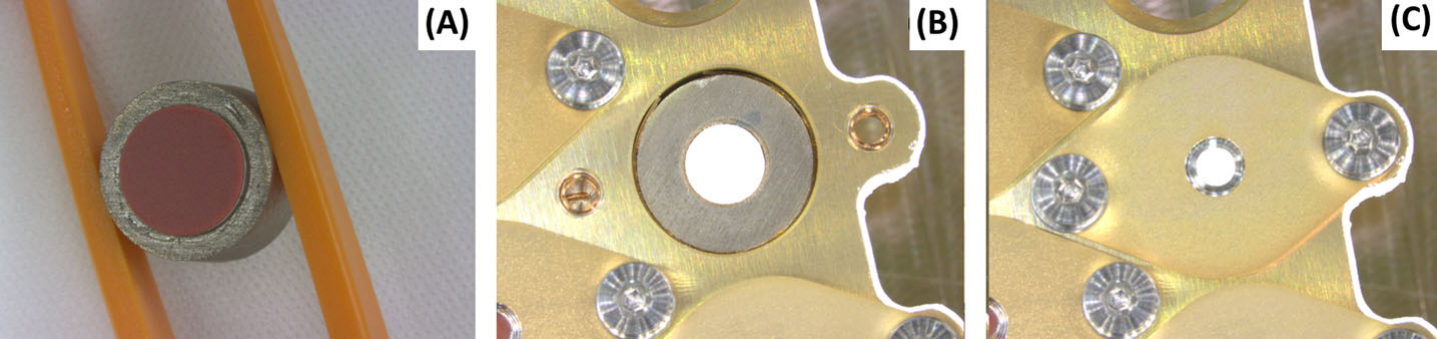
Assembly process of the passive samples with magnets. (A) is the red target inserted within its magnet. (B) The white target plus magnet assembly, upside-down, inserted into the sample holder. The fixation lid on the back of the SCCT (C) applies the designed preload to the target, due to the enclosed washer and spring (not shown). The center of the target can be seen through a hole in the lid (Fig. 7)

The mass budget available for the samples was agreed as part of the preliminary design definition of the SCCT; this was in a much earlier stage of the development than the final definition of the samples. However, including some margins for the mass of the different targets, it was estimated, during the preliminary design of the SCCT, that up to 75% of the mentioned mass budget could be allocated to the holder. The structure of the holder is made of Aluminum 7075-T7351. The overall size is 100 mm × 95 mm, plus an extra 15 mm protrusion for the Ti plate (Fig. 10). The overall thickness is 17.45 mm. Targets 1.1 to 5.6 have 12 mm diameter dedicated holes, including a 1 mm clearance around each sample to cope with manufacturing tolerances of the targets and the Coefficient of Thermal Expansion (CTE) mismatch between the holder and the samples. The targets are inserted from below and held in place by means of a spring-loaded system underneath the samples, which press against a lip at the top. The exposed surface of the samples in the direction of the Mast Unit is 8 mm diameter for each target. Inside their cylindrical enclosures, samples are protected at their base by a custom washer that distributes the load from a wavespring held in tension against the bottom covers. Lateral displacements are limited by friction on the top and bottom of the samples. A cross section for the passive samples (#1.1 – #1.5) is shown in Fig. 8. Following an idea by Madsen et al. (1999) for the Mars Pathfinder lander, a Kapton^®^ shim is wrapped around the magnets to serve as cushion in case of lateral impacts. The other samples (#1.6 to #5.6) use a similar design to that shown in Fig. 8, adapted to their size, and without the ring magnets and Kapton^®^ shims, as shown in the integration process in Fig. 11, and described in Fig. 9. The risk of damage for these samples due to lateral impacts is lower than in the case of passive samples, as the mass, hence the inertia, is lower, and the geological samples are not as brittle as the sintered magnets. The fixation of the whole stack to the holder is performed by means of individual lids fastened to the main structure with self-locking inserts. The purpose was also to minimize the use of glues that are potential sources of volatiles. In addition to 28 cylindrical boreholes to house standards (Fig. 2), the design of the holder was completed with a shallow 1.15 mm deep borehole made from the top side, in which to glue the diamond (#0.1), and four threaded holes to fasten the Ti plate (#0.2) and upper plate that carries the geometric targets (#0.3/04/05) and the Martian meteorite (#0.8). The sample holder is painted white (emissivity of 0.87 and absorptivity of 0.17, target #0.6) with a dark area around the white sample painted with Aeroglaze Z306 (absorptivity > 0.95 and emissivity of 0.9, target #0.7). This provides an abrupt contrast between the passive target and the holder, see element 0.7 in Fig. 2. The inner surfaces were coated with class 3 chromate conversion to prevent corrosion. Reflectance spectra of #0.6 and #0.7 are shown Fig. 5.

The final mass of the holder is 248.5 g, of which 140 g are used by the holder’s main body. This allowed a ruggedized structure around the targets, resulting in a completed assembly with a first normal vibration mode over 2 kHz, which was the qualification level, meaning that the first mode is not expected to be excited, avoiding resonances.

The sample holder is at the rear of the Perseverance rover, on the right side facing in the forward direction (Fig. 1). A bracket raises the sample holder above the rover deck. The original requirement was to be at a distance between 1.5 m and 1.7 m from the Mast-Unit external face. However, the Mast Unit was found to have a back reflection of the infrared laser beam that could damage the secondary mirror when focused at 1.56-1.61 m. The bracket moved the SCCT distance from the middle to the edge of this range. The final range is 1.544 m – 1.567 m, depending on the targets. This is within 2 cm of the distance of Curiosity’s targets to ChemCam, which allows direct comparison of the data. To avoid laser reflection toward the Mast Unit, the sample holder is mounted on the rover such that the angle between its surface normal and the SuperCam boresight varies from 11 to 14 degrees, depending on which individual target is considered. The structure that holds the alumina plates and the meteorite is further tilted upward to give a final angle of ∼6° from normal to the instrument boresight.

After a few iterations with the project, we find that this accommodation is clear of a shadow cast by the helicopter communication antenna and other elements on the rover, especially for the geometric targets and passive targets that require direct sunlight.

## SCCT Models, Integration and Tests

The SCCT includes the sample holder and all targets defined in Table 1. Parts of the SCCT were manufactured in different laboratories and were sent to INTA (Instituto Nacional de Técnica Aeroespacial, in Torrejón de Ardoz, Spain) for integration, under the leadership of the University of Valladolid.

### Model Philosophy

Following JPL’s recommendations, a total of six replicates of the individual targets were necessary:

- CU (Calibration Unit): A set of calibration targets that includes all targets being considered for flight (a larger set). These targets were used for calibration and functional tests during SuperCam’s development. They will be re-used during the mission as part of the ground calibration setups. The same characterization requirements applied to these samples and those flying to Mars.
- ETU (Engineering and Testing Unit): A model used for early shock tests, helping the design of the fixation system and validating the different samples to the shock stress. Once the development of the SCCT was completed, some parts of this model were used in the assembly of the engineering model (EM).
- EM (Engineering Model): An engineering model, representative of the final flight model in form, fit and–although it was not required–from the dynamics point of view. It is not representative, however, of the thermal behavior of the model to operate on Mars, as it is not painted, changing its albedo. From the functional point of view, only samples 1.1 to 1.5 and the geometric target (#03/04 and #05) were mounted in this model. It is to be used at JPL with the ground model of Perseverance.
- EQM (Engineering and Qualification Model): this model is highly representative of the flight unit, with minimum differences in the samples’ configuration (explained later) and paint definition in the dark area around sample 1.1 (#0.7). It passed the qualification campaign at higher stress levels than the flight unit. It went through every process used in the flight model, in the order experienced in flight, including the contamination control and planetary protection measures (described in Sect. 4.3).
- FM (Flight Model) and FS (Flight Spare): these models include the final sets of targets. The only exception is the Martian Meteorite (#0.8 in Fig. 2), of which only a piece was available for the FM. Both units were manufactured together, so processes, materials, and workmanship remained the same. They were tested at acceptance levels. The sterilization processes were identical.

**Fig. 12:**
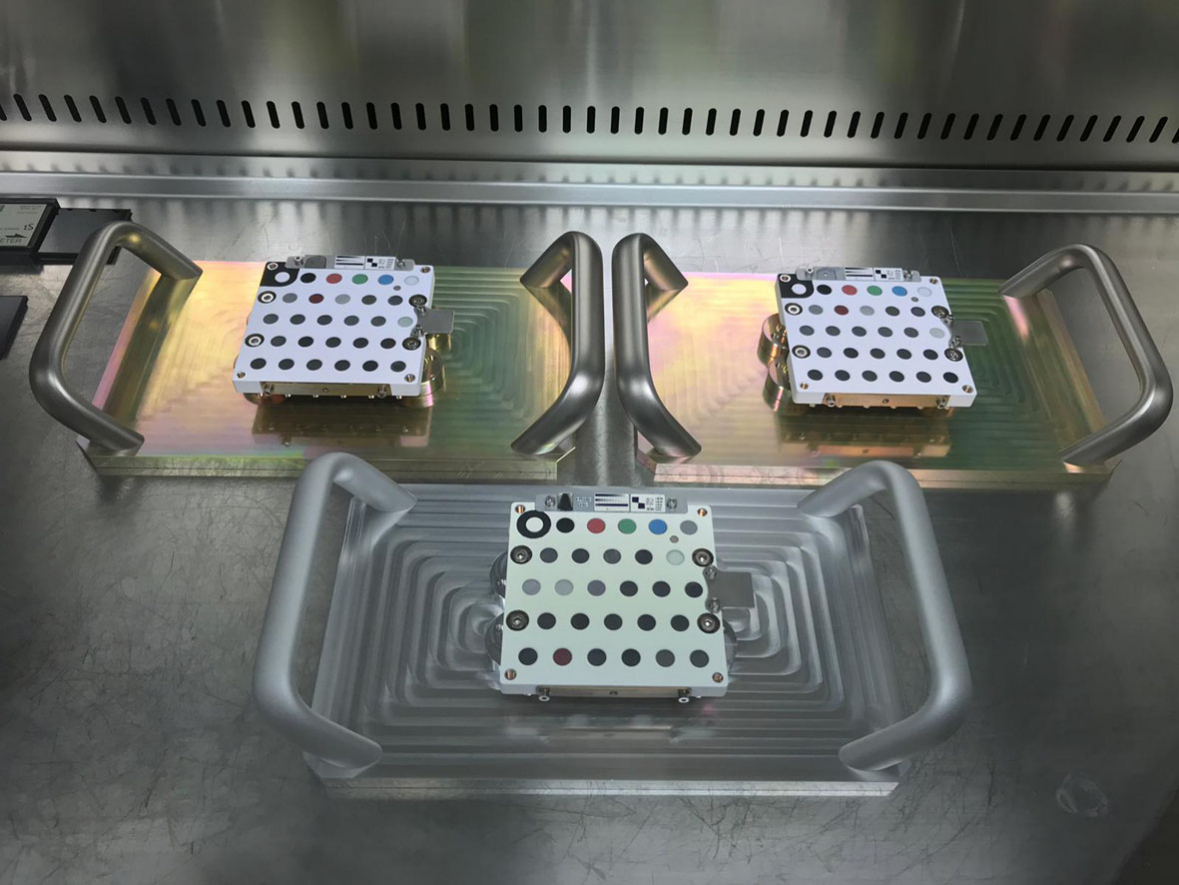
Family picture of three of the SCCT models; FM and FS are on the top; the EQM is at the bottom

The FM, FS and EQM are shown in Fig. 12. If not needed, the FS and EQM will be used with the ground calibration setups, and will remain sterile until then.

### Integration and Tests

All integration activities occurred in a sterile ISO (International Organization for Standardization) 4 laminar flow chamber placed inside an ISO 8 clean room at INTA (Spain), sharing space with the ExoMars/RLS experiment. The cleaning procedures and different bake-outs done prior to integration are described in a later section. The thermal and dynamics tests done in the development (ETU model), qualification (EQM model) and acceptance (FM and FS models) campaigns of the SCCT are described in Table 2.

**Table 2:** Test strategy for SCCT validation and acceptance, including description and rationale per model, except for the EM (no environmental tests)

Test type	Test	Description	Rationale	Models
Dynamics	Sine survey	Low intensity sine vibration up to 2500 Hz	Validation of the hardware natural resonances. Executed before and after each test to monitor the SCCT health.	ETU EQM FM-FS
	Quasistatic loads	Five low-frequency vibration of 72 g bursts.	Verify the survival of the hardware to low-frequency loads received during launch and/or landing.	EQM
	Random vibration	Vibration with a determined frequency response simulating the flight vibration.	Verify the survival of the hardware to the highest loads to be supported by the hardware during flight.	EQM FM-FS
	Shock test	Two three-axis shocks with short decay time. Risk-mitigation: 2000 and 4000 g. Qualification: 3500 g.	Verify the survival of the hardware to the shock produced by pyrotechnic devices to deploy the rover wheels during landing.	ETU EQM
Thermal tests	Thermal Vacuum	Temperature cycling in vacuum (10^−5^ Torr). Qualification: 29 cycles between −135 and 80 °C. Acceptance: 2 cycles between −135 and 70 °C.	Verify the survival of the hardware to the thermal stress produced during cruise and Mars day/night cycles. Acceptance test is shorter to avoid over-stressing the flight hardware.	EQM FM-FS
	Mars pressure	One temperature cycling in Mars pressure conditions (6 mbar of N_2_). Qualification: between −135 and 80 °C. Acceptance: −135 and 70 °C.	Verify the survival of the hardware to the thermal stress in Martian conditions. Test the effect of entry in Mars atmosphere on the hardware due to gas thermal inertia or potential thermal conduction effects.	EQM FM-FS

As introduced in the Models Philosophy, a unit (ETU) was dedicated to make sure that the targets would survive shock during the development test campaign. Three separate shock tests were performed, one 4000 g test at JPL in Pasadena, and 2000 and 4000 g tests at CTA facilities in Vitoria, Spain. The initial test gave a first assessment of the mechanical behavior of most of candidate targets that were finally included in the FM, indicating they were ready to fly. Some additional samples, proposed in a later stage of the development, were tested during the qualification campaign. Improvements were also made in the cleaning procedure to prevent dust ejection from the samples during shocks, and in the assembly of the passive samples, verified during the CTA development shock tests.

**Fig. 13:**
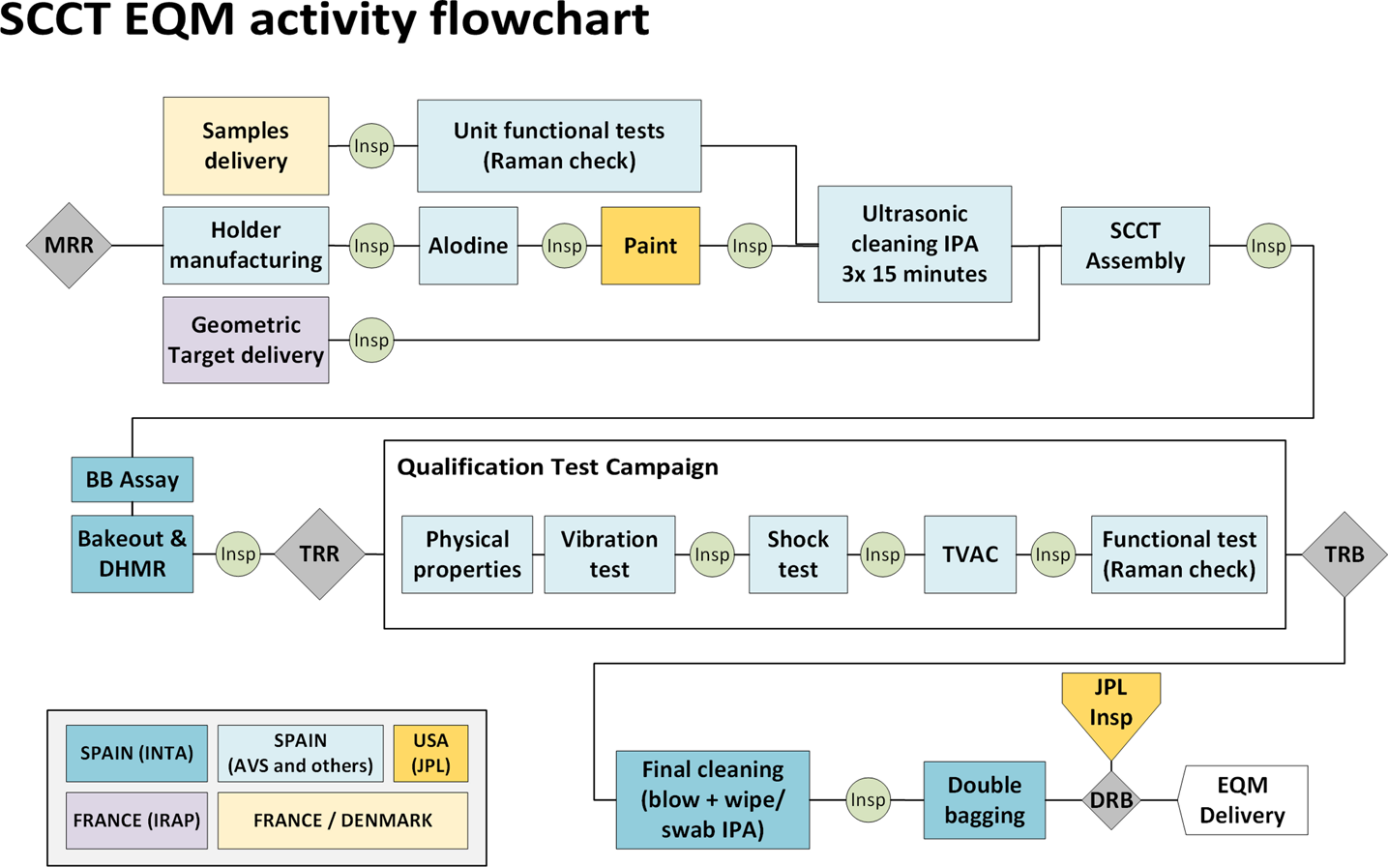
Integration and testing flowchart of the SCCT EQM. Including assembly, qualification campaign and post campaign verification and cleaning. Acronyms are manufacturing readiness review (MRR), isopropyl alcohol (IPA), breadboard (BB), dry-heat microbial reduction (DHMR), test readiness review (TRR), thermal vacuum test (TVAC). Other acronyms are given in the main text

The most complete set of tests at the most representative levels were performed on the EQM during the qualification campaign. The qualification tests were done in a test-as-you-fly configuration, in the same order as for the mission. Figure 13 is an overview of the EQM development scheme. Hence, bakeout/sterilization was performed before environmental tests. The highest temperature faced by the hardware occurred during DHMR, and sensitive parts such as glues needed to be validated by this process. Once the hardware was validated through this process using the EQM, DHMR on the FM was done right before delivery, to keep the hardware as clean as possible in our last access. All qualification tests and acceptance tests to verify the design and workmanship were done in ISO 8 or better (ISO 5 for the FM), with contamination witnesses always present. Dynamics tests of the qualification campaign were done at CTA (Vitoria, Spain), and thermal tests in Alter facilities (Madrid, Spain). The whole acceptance campaign was done at Alter for the FM and FS, as a pyroshock test was not required. This last test is included in qualification campaign to demonstrate the withstanding against shocks but provides no information in terms of workmanship check (main purpose of the acceptance campaign), in addition, a shock test could overstress the flying hardware and mean a risk.

Dynamics tests at qualification levels with the EQM revealed no issue, e.g., no damage to the hardware. The combination of these tests with development tests allowed us to test several candidate geological samples. Two exceptions were not tested before their integration into FM: the Shergottite from ChemCam (target #0.8), and the PET (target #1.6), whose mechanical properties were not deemed a risk.

The thermal tests completed the qualification campaign. Later inspections showed that the hardware complied with the mission requirements. Sensitive areas such as glued parts or paints showed no damage from thermal cycling.

### Contamination Control and Planetary Protection

The RWEB (Remote Warm Electronics Box) window in the Mast Unit (Maurice et al. 2020, this issue) and the SCCT are the only parts of SuperCam directly exposed to the Martian environment, making it critical to keep them clean and sterile. Key and driving requirements applicable to the SCCT are: Particulate Contamination (PAC) < 85 ppm, Molecular Contamination (MOC) < 100 ng/cm^2^, and Bioburden (BB) < 1000 spores/m^2^. To achieve these goals, a series of preventive practices were established during the development of the instrument, as stated in SuperCam assurance and contamination control plans. Cleanliness principles were the following:

- All parts cleaned three times for 15 minutes in ultrasonic baths of isopropyl alcohol;
- Assembly, Integration and Test (AIT) activities occurred in controlled environments (see previous section), with permanent molecular contamination witness tray and a particle fallout witness (PFO);
- Storage of the SCCT in double bags flushed with nitrogen;
- Remove Before Flight (RBF) cover and a transportation fixture to prevent scratched or damaged surfaces during handling;
- Several separated bake-outs and final DHMR were done (see Table 3) to prevent volatiles and cross contamination.

**Table 3:** Parameters of the different bake-outs that were part of Contamination Control (CC) and Planetary Protection (PP) procedures for the SCCT

Parts	Temp. (°C)	Duration (hours)	Rationale
Passive samples (#1.1 to #1.5)	116	48	Curing of glue, removing adhesive volatiles
Geological samples (#2.1 to #5.6)	120	120	Cleaning procedure after characterization
PET sample (#1.6)	120	72	Removing volatiles
Remove Before Flight (RBF): assembly and handling fixture	110	48	Final steps of cleaning procedure
SCCT (whole system)	115	120	Subsystem level DHMR, prior to delivery

The DHMR process and bakeouts were designed according to ECSS (European Cooperation for Space Standardization) rules, using the lowest temperature (plus margins), in the case of DHMR, to avoid damage to the glued parts. The temperature reached in this process is much higher than the SCCT will face during the mission (80 °C was the maximum temperature for qualification), meaning that any possible volatiles that could be a concern during the mission were removed in this step.

As a general procedure, both witness trays were kept close to the SCCT and exposed every time the hardware was exposed. The only exception was the thermal test of the qualification and acceptance campaigns, as only the molecular witness was introduced in the thermal chamber with the SCCT. A final, and conservative, increase of 20 ppm was summed to the actual measurement of the PFO, raising the estimate from 26 ppm to 46 ppm, still well below the 85 ppm requirement. The MOC witness was unique during the whole qualification campaign, including integration and acceptance tests. Measurement yielded contamination levels of 37 ng/cm^2^, below the 100 ng/cm^2^ requirement. The bioburden was assayed several times along the AIT process, especially on the handling areas of the unit, which had been operated repeatedly. All the assays provided counts of zero spores. Considering the minimum of 1 spore as per ECSS, internal surfaces had a bioburden of 150 spores/m^2^ (requirement < 1000 spores/m^2^), while the external surfaces had a total of 500 spores/m^2^ (requirement < 300 spores/m^2^) before DHMR. The DHMR done after the assays resulted in a reduction of four orders of magnitude in these values, meeting all Planetary Protection requirements with margin.

## Conclusions

SuperCam carries a complex set of calibration targets, in line with the ambitions of the instrument. It provides basic references for each measurement technique. Science objectives required the implementation of 23 geological samples, 5 reflectance standards, one organic sample, a diamond, and 4 calibration targets for imaging. For public outreach, it carries a sample from Mars that will return home.

The sample selection has been a long process with participation of the whole science team (see details in Cousin et al., in preparation). The main challenge was to ensure that chemical, mineralogical, and structural properties of the geological samples and passive targets, remain constant along the qualification path. Their composition and homogeneity were analyzed with various techniques, such as LIBS, Raman, and XRF (see Madriaga et al., in preparation). For the sample holder, the difficulty was the mounting system designed to protect brittle samples, avoiding rotation and absorbing extreme mechanical loads.

The SCCT is directly exposed to the Martian environment, making it critical to keep it clean and sterile. Constant attention to contamination control and planetary protection was applied throughout the development of the project. Before delivery, DHMR (115 °C, 120 hr) was applied to the whole SCCT. Resulting contamination and bioburden levels were well within requirements.

The hardware that is mounted on Perseverance’s deck was tested at JPL; some preliminary measurements on the SCCT were be acquired by the instrument (Wiens et al. 2020, this issue). The FM SCCT fulfills the needs of SuperCam’s science team, meeting all the technical requirements and making possible the in-situ calibration of the five analytical techniques in SuperCam.

## Data Availability

Not applicable.
